# Supplementary material for: Diurnal oscillations in gut bacterial load and composition eclipse seasonal and lifetime dynamics in wild meerkats
Source: Nat Commun. 2021 Oct 14;12:6017. doi: 10.1038/s41467-021-26298-5 (PMC8516918; doi:10.1038/s41467-021-26298-5)
Supplement: Supplementary file 1 — Supplementary Information [file 41467_2021_26298_MOESM1_ESM.pdf]

Supplementary Materials for

Diurnal oscillations in gut microbiome load and composition eclipse seasonal and lifetime dynamics in wild meerkats, *Suricata suricatta*

Risely, A.*, Wilhelm, K., Clutton-Brock, T., Manser, M.B., and Sommer, S.

Correspondence to: alice.risely@uni-ulm.de

This file includes:

Supplementary methods 1
Supplementary figures 1 to 12
Supplementary tables 1 to 4

SUPPLEMENTARY METHODS

Supplementary methods 1

Estimating body condition

Individual meerkats are weighed daily by enticing them onto electronic scales using crumbs of hard-boiled egg. Body condition was represented by residuals from a general additive mixed model (GAMM) predicting weight against age and time of day, using all weight data available for individuals included in this study (n = 234,224 weight measurements from 235 meerkats). Individual ID was included as a random effect. Although age is the major predictor of weight, we included time of day because body mass changes over the day in response to food intake. In almost all cases a weight measurement was taken on the day of sampling, and for a small proportion of samples weight was taken within a 1-3 days of sampling.

Foraging schedule

We reasoned that gut microbiome dynamics is likely to be impacted by daily foraging patterns and therefore aimed to estimate foraging history at the time of sample collection. Meerkats demonstrate relatively predictable daily foraging patterns, with two peaks in foraging intensity – one in the morning and a shorter period in the evening before sunset (Fig. 1a; Doolan and MacDonald, 1996; unpublished data Kalahari Meerkat Project). In the summer, when temperatures reach 40°c during the day, meerkats forage early in the morning and again in the evening, and find shade during the day. In the winter, meerkats maintain this foraging pattern in the morning and evening, yet also forage to some extent during the day (Doolan and MacDonald, 1996). In this study, we calculated mean foraging start and end times per month based on long-term observation data (Fig. S7), and assumed meerkats only foraged during these times. Visual inspection of different social group strategies indicated all groups followed similar foraging schedules across the year. Whilst some foraging is likely to occur outside these times in winter, these represent periods of the most intense foraging and therefore may be expected to be reflected in microbiome dynamics if short-term foraging patterns affect the gut microbiome. From this schedule, we calculated how long each meerkats had been foraging for prior to sample collection. If the sample was collected outside a foraging period (which was a minority of samples), this number was set to zero.

Time spent in the field

We wanted to account for variation in field time to ensure this variable did not bias results. Meerkats are monitored during their active foraging periods, and therefore fieldwork is carried out at set times throughout the year. We therefore calculated mean morning and afternoon fieldwork return times per month (because fieldwork times shift with season to match meerkat foraging patterns) and estimated the number of hours each sample was carried in the field before being frozen.

Weather data

Hourly and daily weather data dating back to 1997, when the earliest sample was taken, was provided by the South African Weather Service. Weather data was collected from the Van Zyl Rus weather station, approximately 25 km away from the study area. We included daily maximum and minimum temperatures, and the temperature at the time the sample was collected. Total rainfall from the month prior to sample collection was also calculated. Missing weather data after 2009 (n = 30) was replaced by weather data collected by the Kalahari Research Centre, whilst missing weather data prior to this point (n = 50) was replaced with mean values for that calendar day calculated across 20 years.

SUPPLEMENTARY FIGURES

Supplementary figure 1

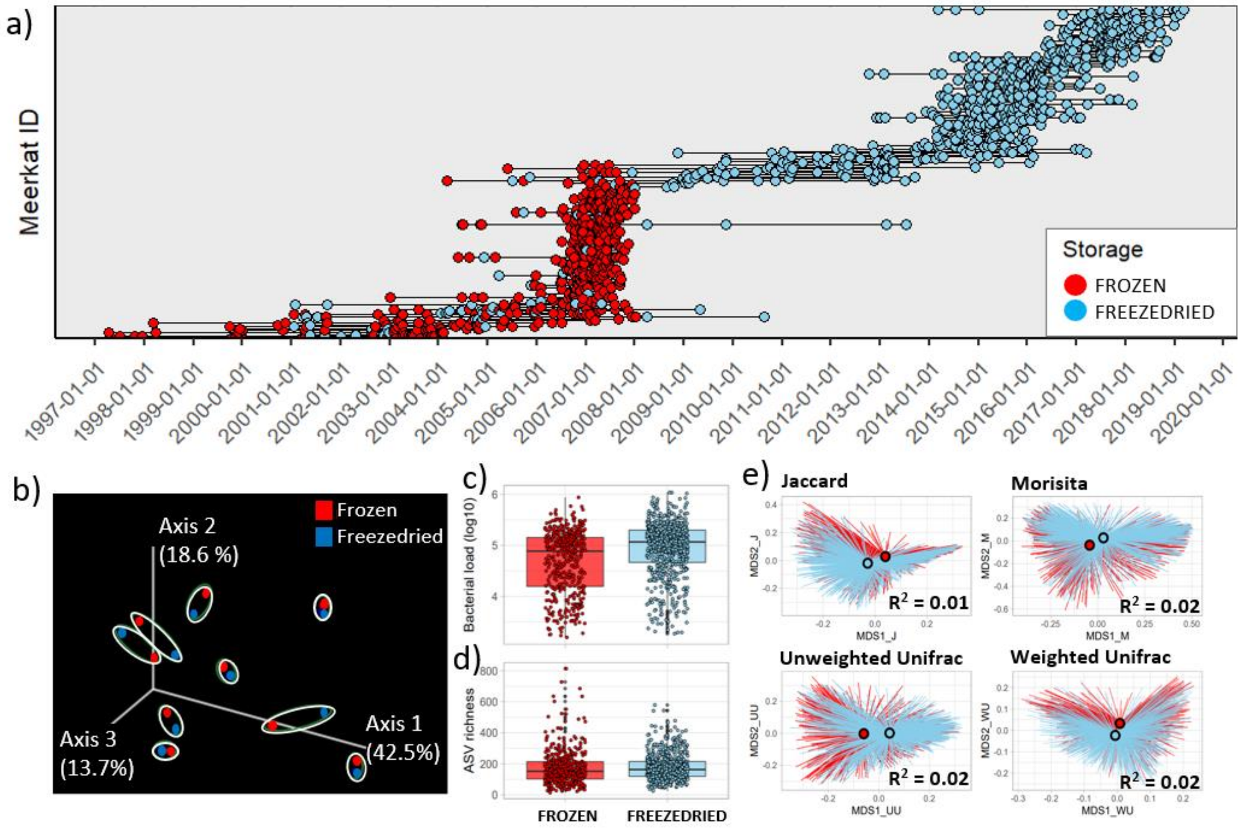

Supplementary figure 1) Effect of storage method on the microbiome. a) Sample timeline coloured by sample storage method (n = 1109). Samples are connected by host ID on the y axis; b) Beta diversity clustered by identity not storage method in an experiment to test the effect of sample storage on nine samples collected from nine captive meerkats (n = 18); c) Bacterial load of frozen and freeze-dried samples (n freezedried = 648, n frozen = 461). Boxplots show the median and lower and upper quartiles; d) ASV richness of frozen and freeze-dried samples; and e) Beta diversity spider plots of samples analyzed in this study (measured with Jaccard, Morisita, Unweighted Unifrac and Weighted Unifrac distances) coloured by storage method. Group centroids are represented by circles. R^2 values from PERMANOVAs. Data to generate this figure is available in the source data file.

Supplementary figure 2

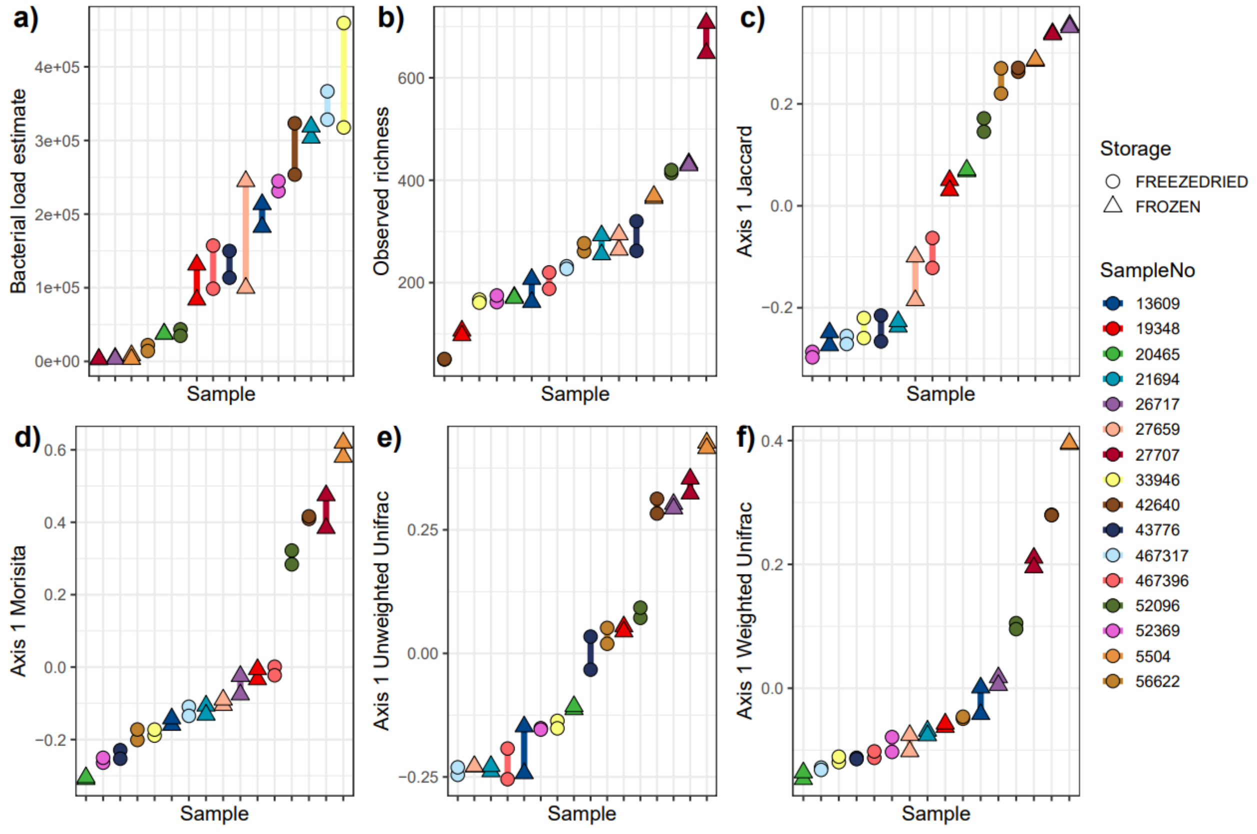

Supplementary figure 2) Technical replication across 16 samples (eight frozen, eight freeze-dried) when measured by a) bacterial load; b) observed ASV richness; c) axis 1 of a Jaccard MDS ordination; d) Axis 1 of Morisita ordination; e) Axis 1 of an Unweighted Unifrac MDS ordination; and f) axis 1 of a Weighted Unifrac MDS ordination. Points are coloured by sample ID and shaped by whether they were frozen or freeze-dried. Data to generate this figure is available in the source data file.

Supplementary figure 3

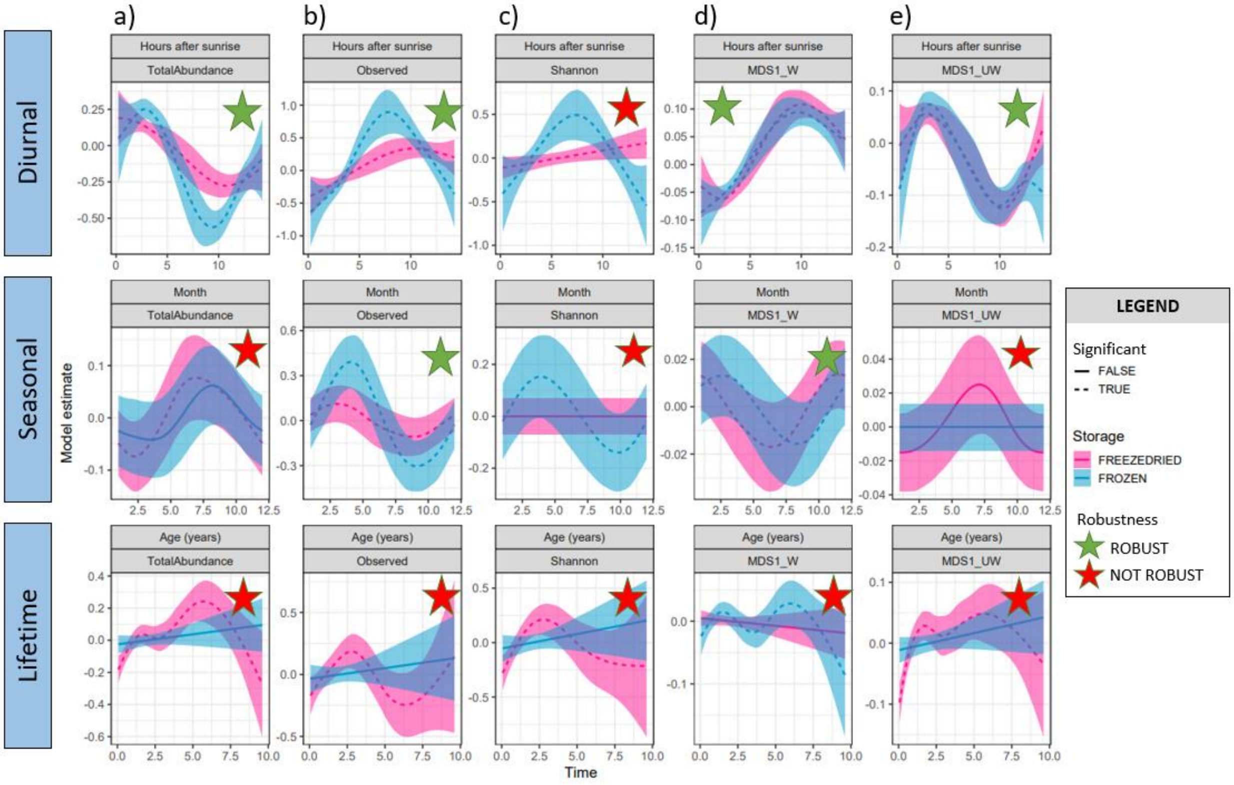

Supplementary figure 3) Trends and 95% CIs in bacterial load and four measures of diversity across diurnal (top), seasonal (middle), and lifetime (bottom) temporal scales, split by storage; a) Bacterial load; b) Observed ASV richness; c) Shannon index; d) axis 1 of a Weighted Unifrac MDS ordination; e) axis 1 of an Unweighted Unifrac MDS ordination. Trends are coloured by storage, and shaped by whether the trend was significant. Stars mark trends that show similar patterns when split by storage (green star) and those that do not (red star). Note that the y axis is scaled to each plot. Data to generate this figure is available in the source data file.

Supplementary figure 4

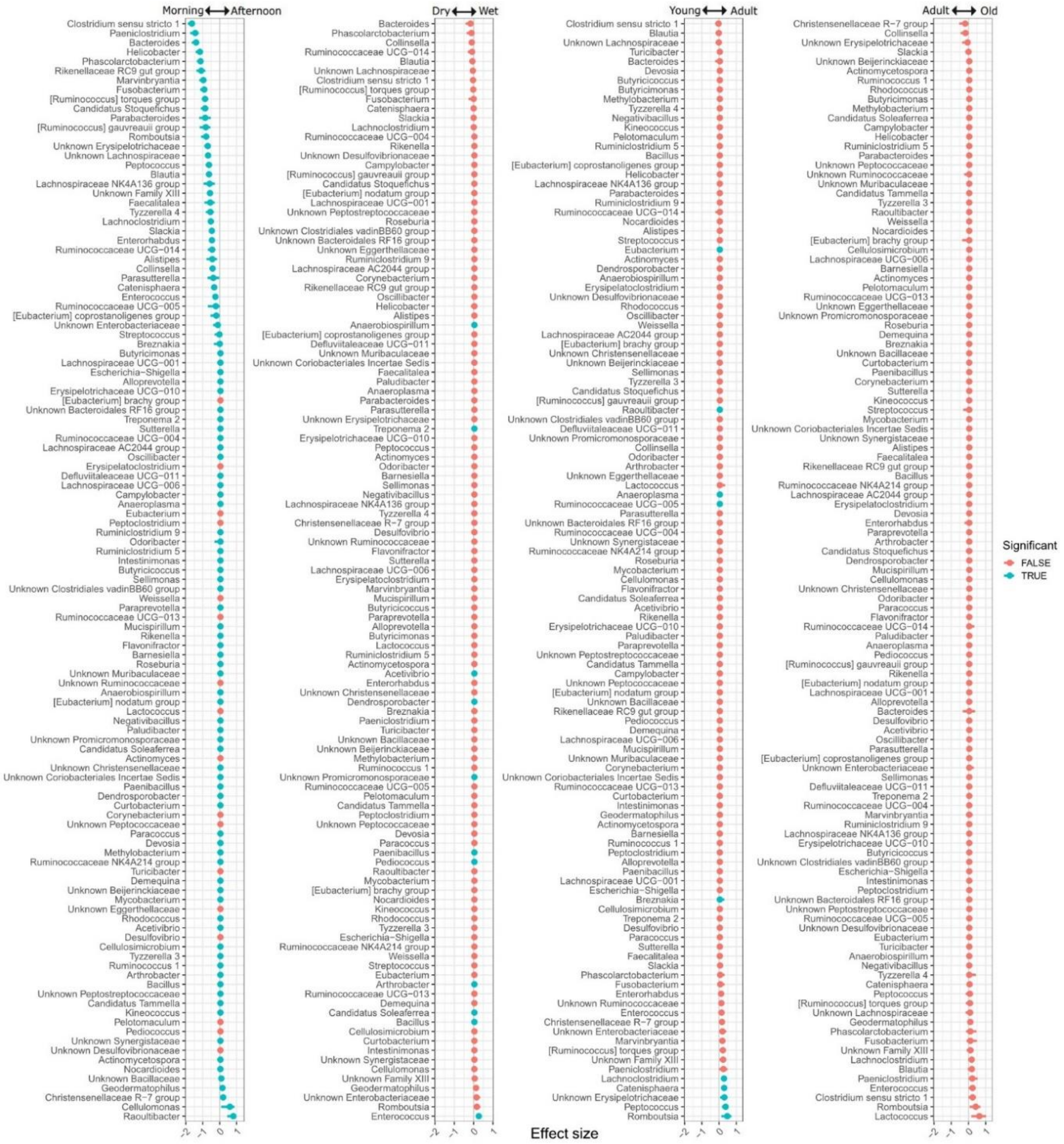

Supplementary figure 4) Differential abundance analysis (two-sided Wilcoxon test) for 117 genera with over 15% prevalence across samples (> 166 samples) between morning/afternoon (n = 743/366), wet season/dry season (n = 691/418), young/adult (n = 385/236), and adult/old (n = 236/97). Estimates and 95% confidence intervals are shows. Blue and red points indicate a significant ($p < 0.05$) and non-significant difference, respectively. P values have been adjusted for multiple testing using the Bonferroni correction. The x-axis is standardized across plots so that effect size are comparable. Data to generate this figure is available in the source data file.

Supplementary figure 5

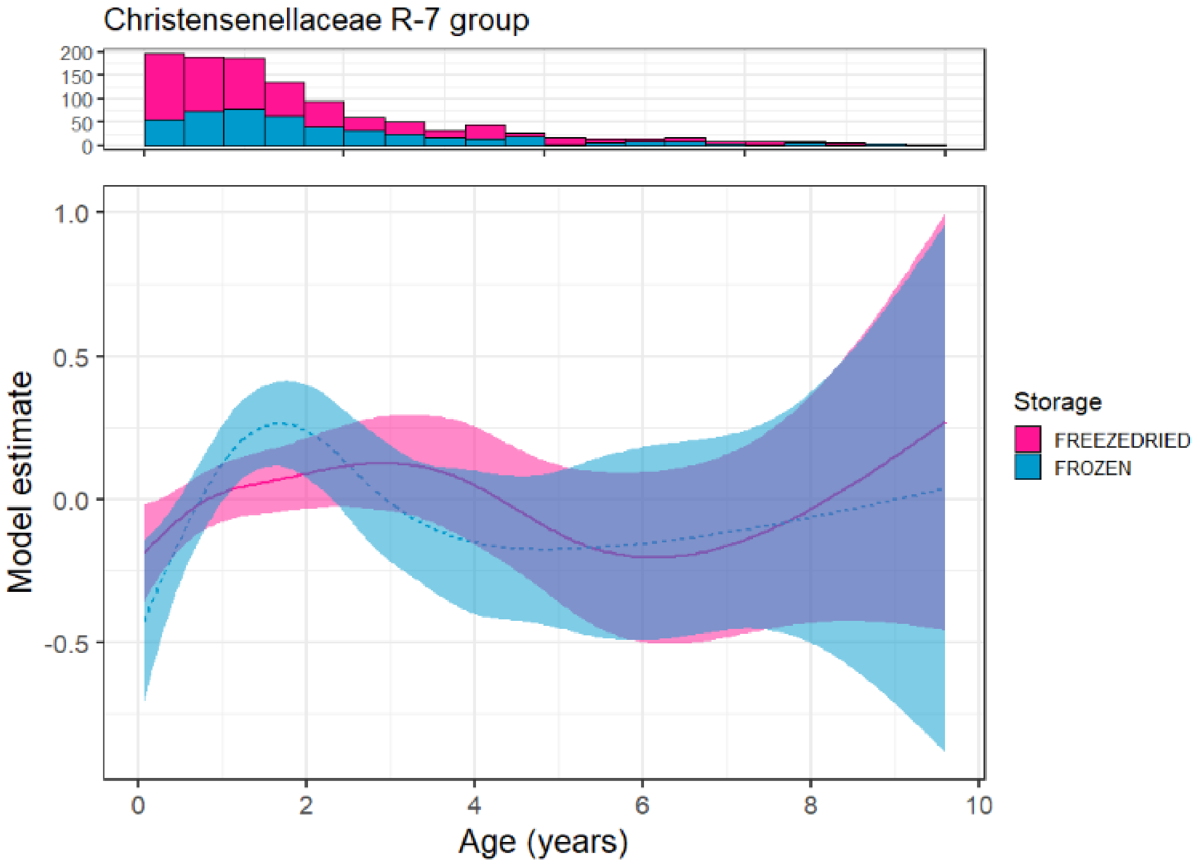

Supplementary figure 5) Changes to *Christensenellaceae R7 group* over meerkat age with 95% CIs, with trends split and coloured by storage. Sample distribution by storage is indicated in the histogram. Data to generate this figure is available in the source data file.

Supplementary figure 6

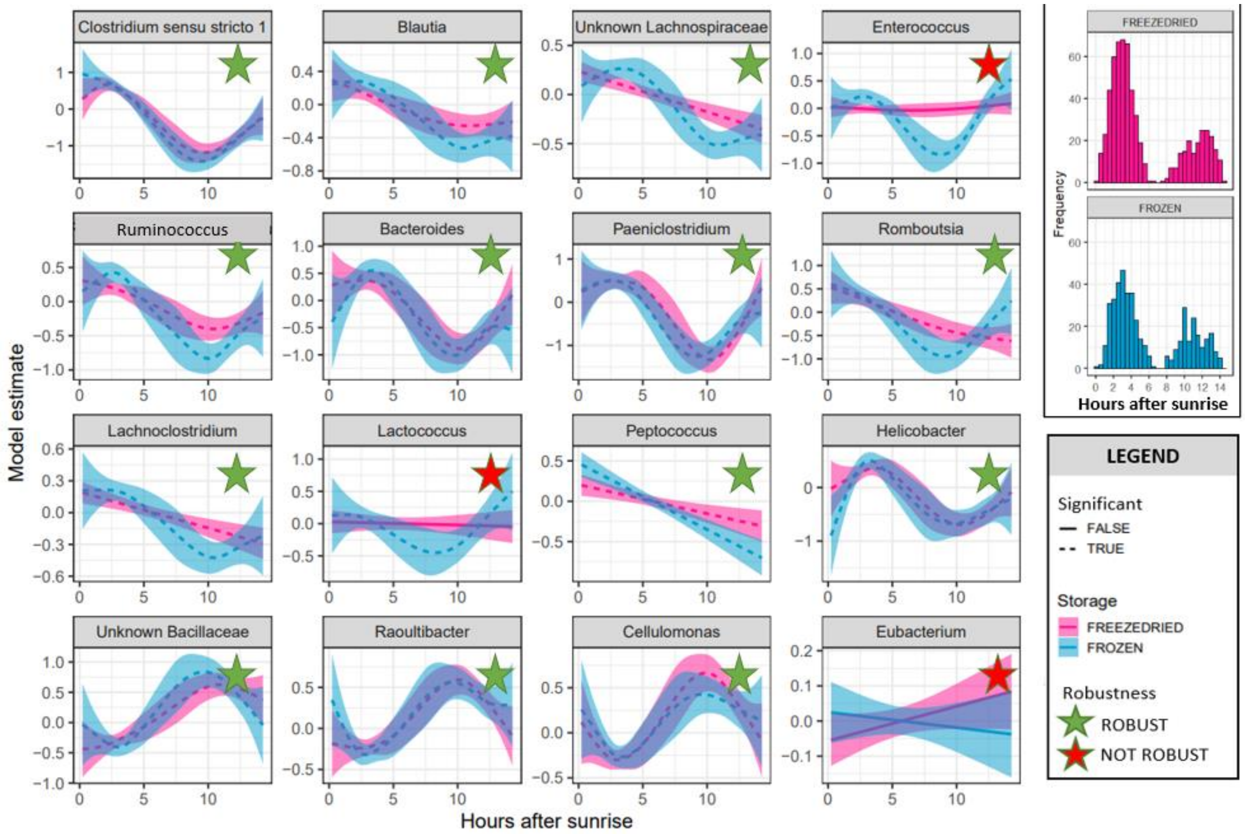

Supplementary figure 6) Trends and 95% CIs in the abundances of 16 core taxa across the day, split by storage, with a histogram showing sample distributions on the right. Trends are coloured by storage, and shaped by whether the trend was significant. Stars mark trends that show similar patterns when split by storage (green star) and those that do not (red star). Data to generate this figure is available in the source data file.

Supplementary figure 7

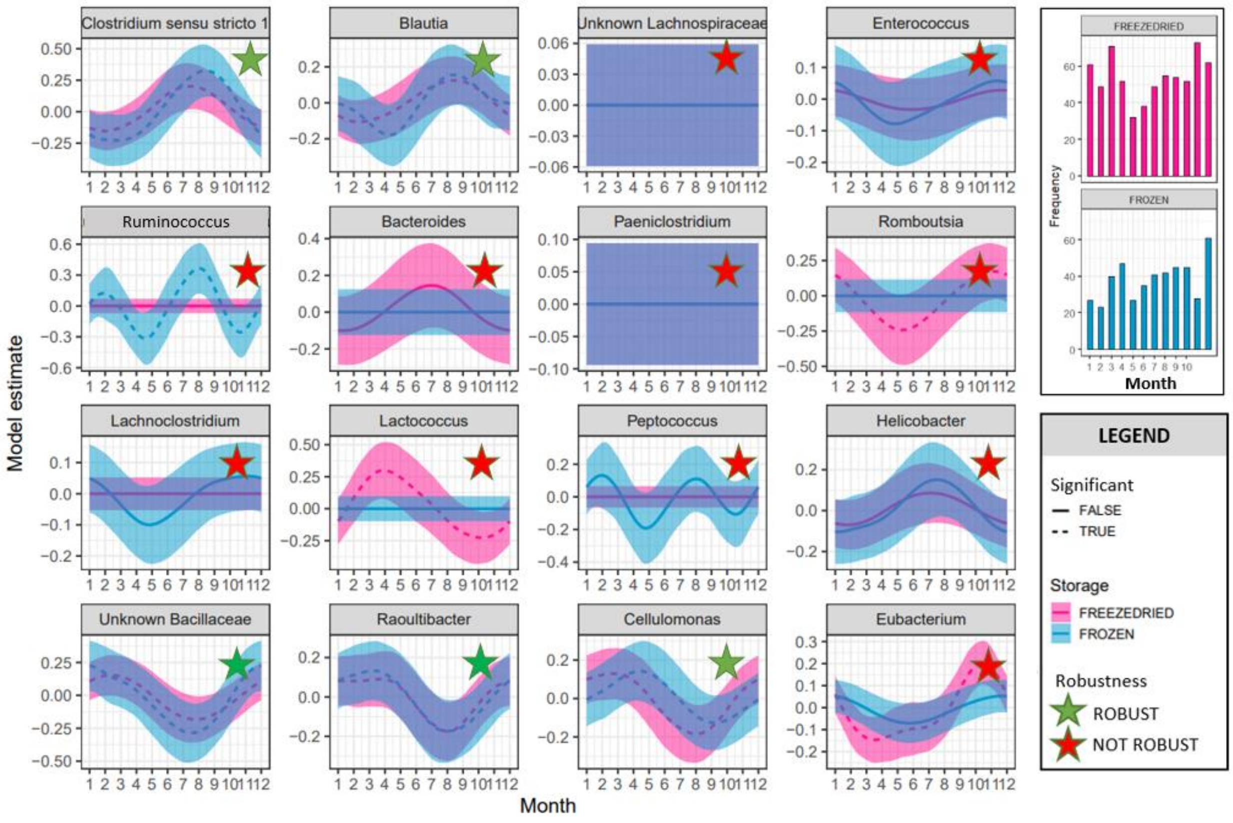

Supplementary figure 7) Trends and 95% CIs in the abundances of 16 core taxa across the year, split by storage, with a histogram showing sample distributions on the right. Trends are coloured by storage, and shaped by whether the trend was significant. Stars mark trends that show similar patterns when split by storage (green star) and those that do not (red star). Data to generate this figure is available in the source data file.

Supplementary figure 8

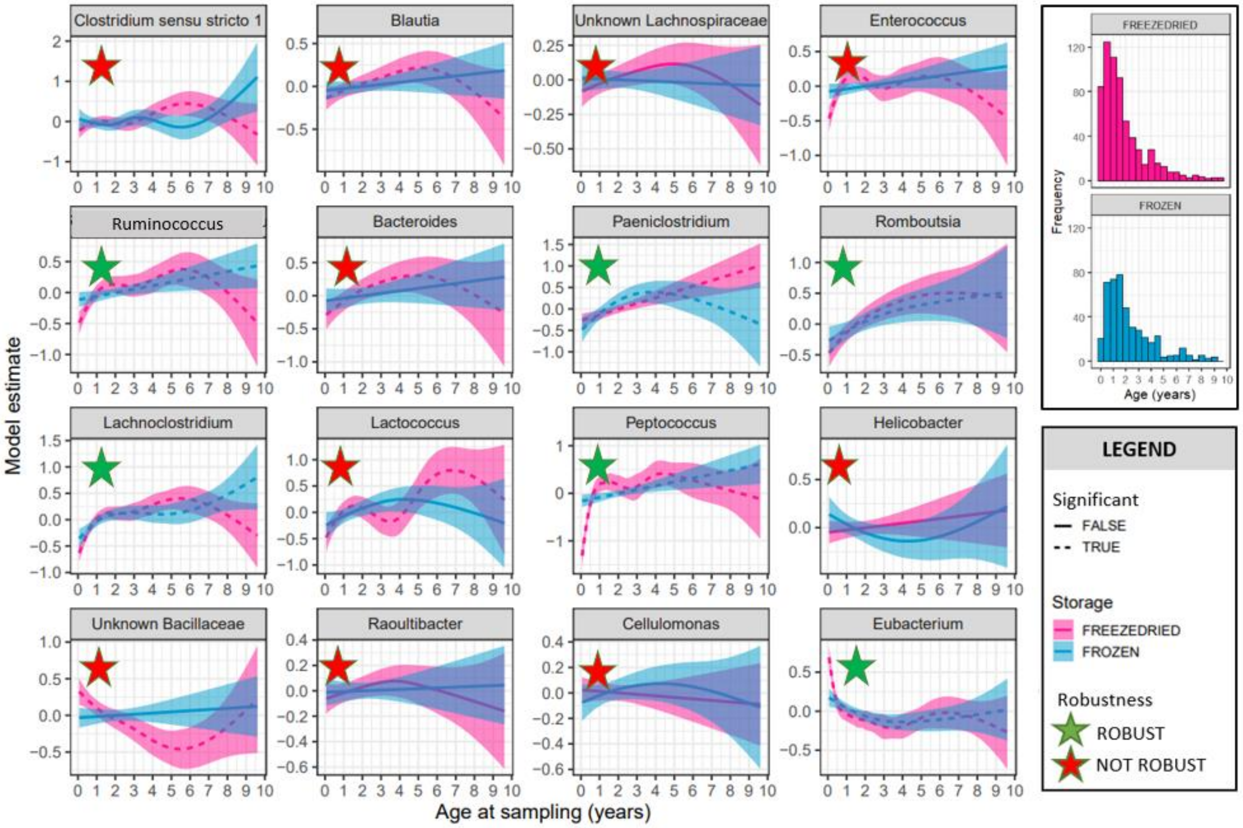

Supplementary figure 8) Trends and 95% CIs in the abundances of 16 core taxa across meerkat life, split by storage, with a histogram showing sample distributions on the right. Trends are coloured by storage, and shaped by whether the trend was significant. Stars mark trends that show similar patterns when split by storage (green star) and those that do not (red star). Data to generate this figure is available in the source data file.

Supplementary figure 9

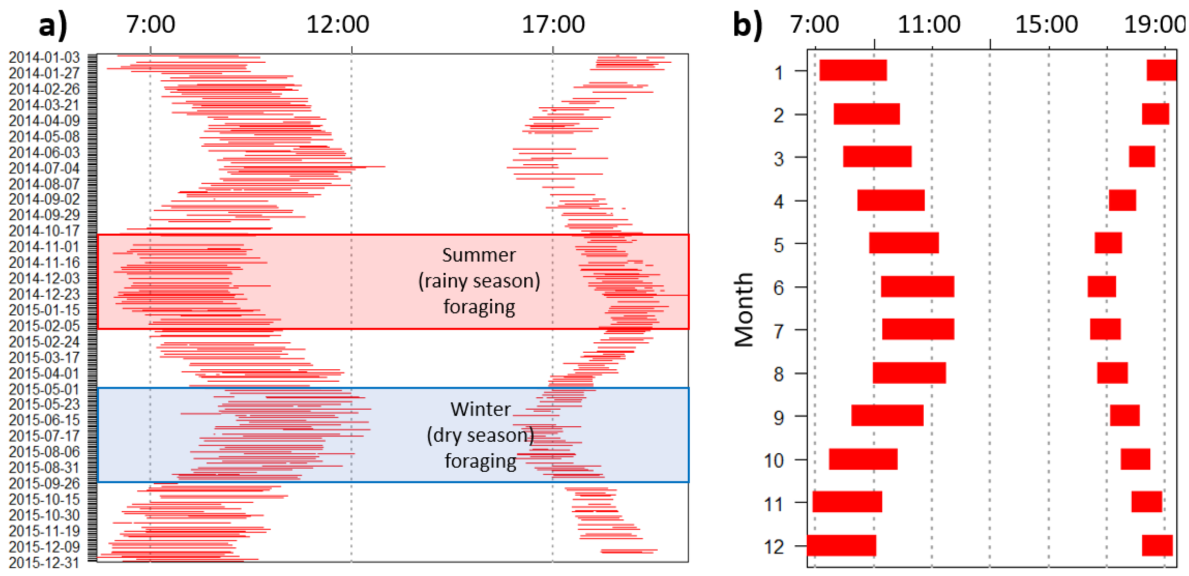

Supplementary figure 9) Meerkat foraging schedule. a) Recorded foraging times of the social group Rascals between 2014 and 2016 as an example of how foraging schedule was calculated, with peak summer (wet) and peak winter (dry) seasons highlighted; b) Mean foraging times per month calculated from 20 years of foraging surveys. This schedule is used to calculate estimates of foraging history prior to faecal sample collection. Note that during peak winter meerkats may forage outside of these times, and therefore the schedule represents an approximate estimate for when meerkats forage most intensely.

Supplementary figure 10

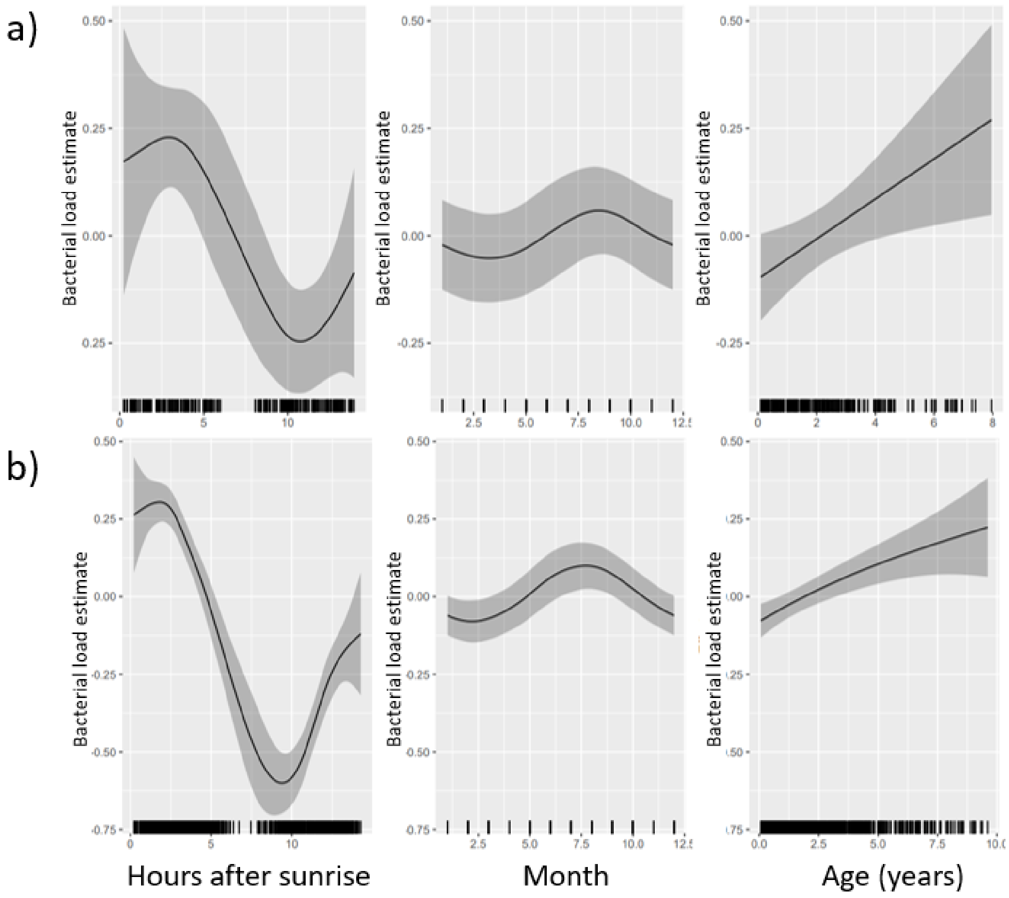

Supplementary figure 10) Sensitivity analyses of bacterial load models. Estimates and 95% confidence intervals for bacterial load when a) 20 randomly selected samples are sub-sampled per hour interval across the day (n = 240); and b) when methodological variables are excluded (n = 1109).

Supplementary figure 11

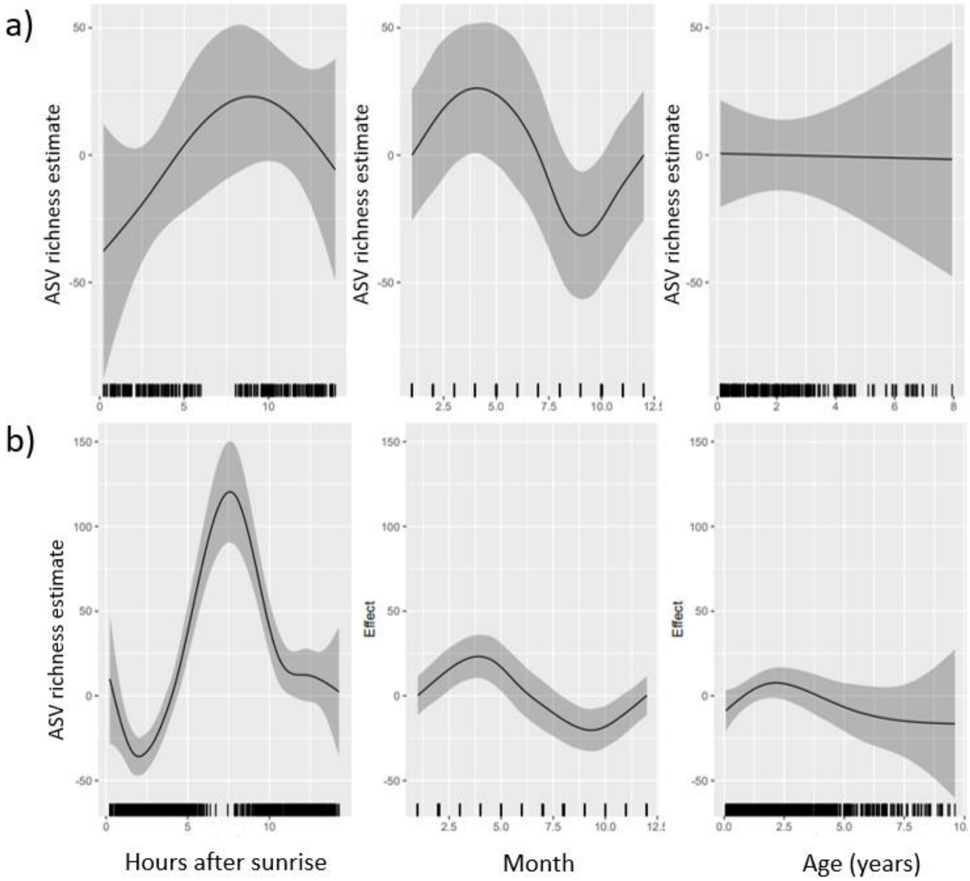

Supplementary figure 11) Sensitivity analyses of models predicting ASV richness. Estimates and 95% confidence intervals for alpha diversity when a) 20 randomly selected samples are sub-sampled per hour interval across the day (n = 240); and b) when methodological variables are excluded (n = 1109).

Supplementary figure 12

a) Principal component loadings

Variable	PC1 (63%)	PC2 (24%)	PC3 (9%)	PC4 (4%)
Rainfall	0.23	**0.96**	0.14	0.13
Temp at sampling	0.53	-0.22	**0.79**	-0.20
Max. temp	**0.58**	-0.20	-0.25	**0.75**
Min. temp	0.57	0.00	-0.54	-0.62

b)

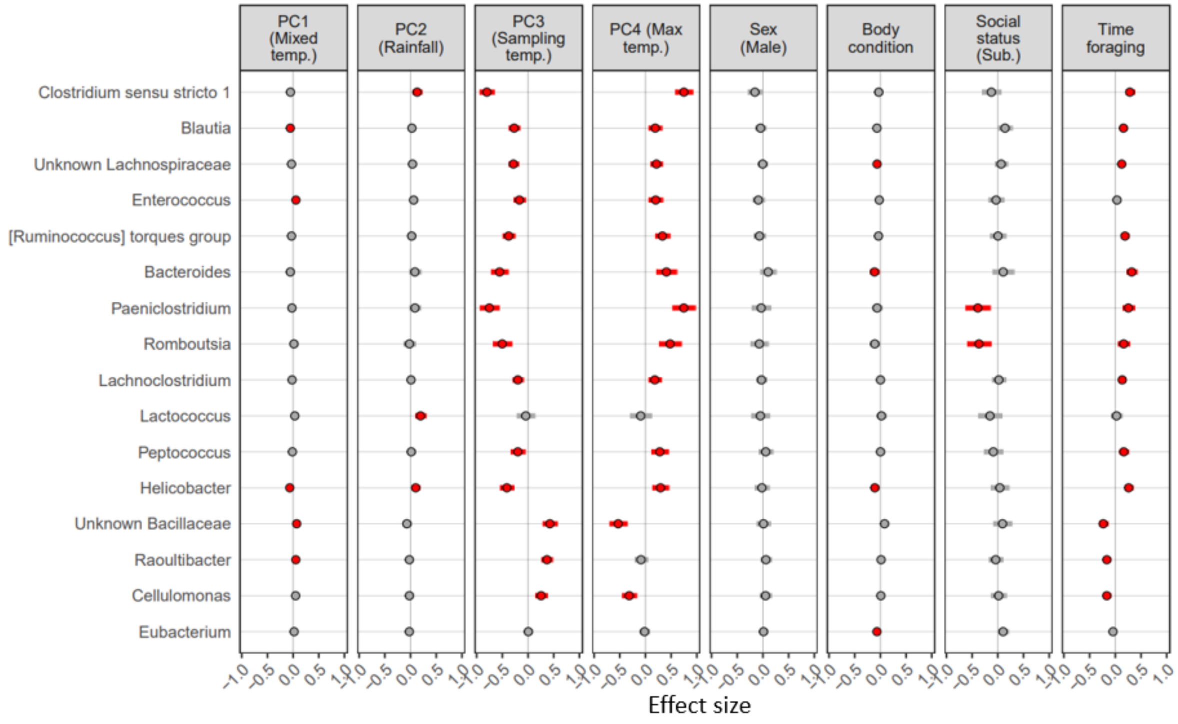

Supplementary figure 12) To account for co-correlation of climate variables, we repeated the GAMM analysis testing for mechanistic effects on genera abundances using principal components from a PCA on climate variables (n = 1109). a) PCA loadings and contributions, with largest contributors highlighted in bold, and b) model estimates and 95% confidence intervals when climate variables are replaced by principal components. Significant associations are coloured red. Data to generate this figure is available in the source data file.

SUPPLEMENTARY TABLES

Supplementary table 1

	VARIABLE	DESCRIPTION
**TEMPORAL**	Hours after sunrise	Time of day sample was taken, with reference to sunrise time on the day of collection
	Month	Month the sample was taken
	Age	Meerkat age in years at the time the sample was collected
**BIOLOGICAL AND BEHAVIOURAL**	Meerkat ID	ID of the sampled individual
	Sex	Sex of the sampled individual
	Body condition	Residuals from a model predicting weight against age and time of day (meerkats are weighed both in the morning and evening) at the time of sampling
	Social status	Whether the individual was dominant or subordinate at the time of sampling
	Social group	Social group membership at the time of sampling
	Time foraging	Number of hours since the last predicted morning or afternoon foraging session started. Foraging periods were estimated per month from foraging records
**ENVIRONMENTAL**	Rainfall	Sum rainfall over the previous month prior to sample collection
	Min. temp.	The minimum night-time temperature of the night before sample collection
	Max. temp.	The maximum day temperature on the day of sample collection
	Sampling temp.	The temperature at the time of sample collection, rounded to the nearest hour
**METHODS**	Storage	Whether the sample was stored frozen or freeze-dried. Samples collected prior to 2008 were frozen at -80°C, whilst from 2008 samples were freeze-dried and stored at room temperature
	Sampling depth	The sequencing depth of the true microbiome, with internal standards excluded
	Sequencing run	Illumina sequencing run (1-4)
	Field time	Estimate of the number of hours the sample was in the field before being frozen

Supplementary table 1) Variables included in this study and their description.

Supplementary table 2

Smooth term	edf	ref.df	statistic	p.value
**s(Hours after sunrise)**	**4.64**	**4.6**	**54.36**	**<2e^{-16}**
**s(Sequencing depth)**	**5.11**	**5.1**	**53.71**	**<2e^{-16}**
**s(Age)**	**1.65**	**1.6**	**9.10**	**0.003**
**s(Month)**	**2.06**	**8.0**	**1.05**	**0.007**
**s(Social group)**	**10.20**	**41.0**	**0.61**	**0.001**
s(Meerkat ID)	9.64	234.0	0.04	0.278

Fixed term	estimate	std.error	statistic	p.value
**(Intercept)**	**4.94**	**0.0**	**125.97**	**<2e^{-16}**
**Sequencing run RUN4**	**-0.65**	**0.1**	**-6.26**	**5.5e^{-10}**
**Storage FROZEN**	**-0.12**	**0.0**	**-3.10**	**0.002**
Field time	0.05	0.0	1.74	0.082
Sequencing run RUN3	-0.02	0.0	-0.46	0.642
Sequencing run RUN2	0.00	0.0	-0.06	0.955

Supplementary table 2) Model summary of a GAMM predicting log10-transformed bacterial load. Variables are split into smoothed (non-linear) and parametric (linear) terms, and terms are ordered by their effect size. Edf = estimated degrees of freedom (degree of non-linearity, where 1 represents a linear relationship); Ref.df = reference degrees of freedom; RE = random effect. Significant terms are highlighted in bold.

Supplementary table 3

Smooth term	edf	ref.df	statistic	p.value
**s(Hours after sunrise)**	**3.60**	**3.6**	**20.80**	**$9.27e^{-14}$**
**s(Sequencing depth)**	**3.73**	**3.7**	**11.03**	**$1.60e^{-07}$**
s(Age)	2.68	2.7	3.76	0.072
**s(Month)**	**2.92**	**8.0**	**3.39**	**$1.07e^{-06}$**
**s(Meerkat ID)**	**54.32**	**234.0**	**0.33**	**0.001**
s(Social group)	0.00	41.0	0.00	0.616

Fixed term	estimate	std.error	statistic	p.value
**(Intercept)**	**153.23**	**7.3**	**21.08**	**$<2e^{-16}$**
**Sequencing run RUN4**	**119.10**	**19.1**	**6.23**	**$6.79e^{-10}$**
**Sequencing run RUN2**	**32.70**	**8.4**	**3.88**	**0.0001**
**Sequencing run RUN3**	**25.18**	**8.6**	**2.91**	**0.004**
Storage FROZEN	-8.29	6.6	-1.26	0.209
Field time	1.79	4.8	0.37	0.711

Supplementary table 3) Model summary of a GAMM predicting ASV richness (alpha diversity). Variables are split into smoothed (non-linear) and parametric (linear) terms, and terms are ordered by their effect size. Edf = estimated degrees of freedom (degree of non-linearity, where 1 represents a linear relationship); Ref.df = reference degrees of freedom; RE = random effect. Significant terms are highlighted in bold.

Supplementary table 4

	Term	Df	SumOfSqs	F	R^2	P. value
a) Weighted Unifrac	**Hours after sunrise**	**1**	**3.06**	**49.66**	**0.038**	**0.001**
	**Sequencing depth**	**1**	**1.15**	**18.66**	**0.014**	**0.001**
	**Age**	**1**	**0.33**	**5.28**	**0.004**	**0.001**
	**Field time**	**1**	**0.25**	**3.97**	**0.003**	**0.002**
	**Month**	**1**	**0.19**	**3.08**	**0.002**	**0.003**
	**Sequencing run**	**3**	**0.38**	**2.05**	**0.005**	**0.003**
	Storage	1	0.07	1.17	0.001	0.298
	**Meerkat ID**	**230**	**15.24**	**1.08**	**0.189**	**0.032**
	Social group	37	2.06	0.90	0.026	0.877
b) Unweighted Unifrac	**Hours after sunrise**	**1**	**5.00**	**24.02**	**0.019**	**0.001**
	**Sequencing depth**	**1**	**2.10**	**10.09**	**0.008**	**0.001**
	**Age**	**1**	**1.12**	**5.35**	**0.004**	**0.001**
	**Month**	**1**	**0.49**	**2.36**	**0.002**	**0.003**
	**Field time**	**1**	**0.41**	**1.95**	**0.002**	**0.012**
	**Sequencing run**	**3**	**0.96**	**1.54**	**0.004**	**0.006**
	Storage	1	0.24	1.14	0.001	0.214
	**Meerkat ID**	**230**	**53.20**	**1.11**	**0.205**	**0.001**
	**Social group**	**37**	**8.32**	**1.08**	**0.032**	**0.05**

Supplementary table 4) Model summary of a marginal PERMANOVAs predicting beta diversity based on a) Weighted Unifrac and b) Unweighted Unifrac. Terms are ordered by their effect size (F) and significant terms ($p < 0.05$) are highlighted in bold.
